# Development and Usability Assessment of a Mobile App (Demensia KITA) to Support Dementia Caregivers in Malaysia: A Study Protocol

**DOI:** 10.3390/ijerph191911880

**Published:** 2022-09-20

**Authors:** Nurul Syaireen A. Rashid, Xin Wee Chen, Muhamad Fadhil Mohamad Marzuki, Aseel A. Takshe, Ahmad Okasha, Faridah Maarof, Raudah Mohd Yunus

**Affiliations:** 1 Department of Public Health Medicine, Faculty of Medicine, Universiti Teknologi MARA Sungai Buloh, Sungai Buloh 47000, Selangor, Malaysia; 2 Kedah State Health Department, Simpang Kuala, Jalan Kuala Kedah, Alor Setar 05400, Kedah, Malaysia; 3 Department of Environmental Health Sciences, Faculty of Communication, Arts and Sciences, Canadian University Dubai, Al Safa Street—Al Wasi City Walk Mall, Dubai P.O. Box 17781, United Arab Emirates; 4 Institutional Research and Planning, Canadian University Dubai, Dubai P.O. Box 17781, United Arab Emirates

**Keywords:** dementia caregivers, caregiver burden, digital technology in caregiving, dementia mobile app, mobile app usability

## Abstract

The impact of dementia on caregivers is complex and multi-dimensional. In low- and middle-income settings, caregivers are often left without adequate support, despite their multiple needs. These include health information, caregiving skills, social and emotional support, and access to local resources—all of which can be partially fulfilled by technology. In recent years, mobile apps have emerged and proven useful for caregivers. We found a few existing apps suitable for Malaysian users in terms of affordability and cultural and linguistic compatibility. Our study aims to design a mobile app that suits dementia caregivers in Malaysia and consists of three phases. Phase I is content development that employs Focus Group Discussion (FGD) and Nominal Group Technique (NGT) involving field experts. Phase II comprises a mobile app (Demensia KITA) designed in collaboration with a software developer specializing in mobile health apps. Phase III entails testing the usability of the app using the Malay version of the mHealth App Usability Questionnaire (M-MAUQ). This study protocol elaborates on the rigorous steps of designing a mobile app and testing its usability, along with anticipated challenges. Our protocol will provide insight for future researchers, healthcare providers, and policymakers and pave the way for better use of digital technology in the field of aging and caregiving.

## 1. Introduction

### 1.1. Background

Most people living with dementia (PLwD) are in low- and middle-income (LMIC) countries [1,2], posing a great economic burden to these nations [1,2,3]. Worldwide, dementia has resulted in disability and reliance among older persons [2]. In the context of developing regions, family members are the most common caregivers for dementia patients regardless of their disease stage [4]. Dementia is a general term for memory, thinking, and decision-making impairment that interferes with daily activities, and is not always part of normal aging [5]. Dementia affects approximately 7% of adults aged 60 and older [2,6]. Globally, the number of individuals aged 60 or over is expected to reach 1.4 billion by 2030, and nearly 2.1 billion by 2050 [7]. This rapid increase in older populations could indicate an upward trend in dementia prevalence. Currently, in Asia, the pooled prevalence of all-type dementia for people over 60 years old is 3299 per 10,000 people [8], and the number is expected to rise further by 2030 [2]. In Malaysia, the prevalence of dementia among older adults in 2018 stood at 8.5% [9]. 

There is a general lack of awareness and understanding of dementia, resulting in stigma and barriers to diagnosis and treatment [2]. PLwD typically has concurrent health problems, such as diabetes mellitus and vascular-related diseases [10], which complicate their treatment and lead to high costs of healthcare [3,10]. Dementia brings physical, psychological, social, and economic consequences that affect not only people living with dementia (PLwD) but also their caregivers, families, and society. Studies found that informal caregivers (e.g., family members) spent an overwhelming average of five hours a day caring for people with dementia [2] and are more vulnerable to mental disorders such as depression [11]. This could be attributed to factors such as the lack of a support system, high caregiving strain, insufficient coping skills, inability to access local resources, care recipient behavioral problems, economic burden due to direct medical and non-medical costs, as well as potential income losses [3,12]. 

In recent years, digital technology has become part and parcel of health and the healthcare system, including in the geriatric and caregiving fields. For instance, mobile applications (more commonly known as mobile apps) could improve public health [13] as they have become more visible and popular as tools for disseminating health information, connecting users with surrounding resources, and healthcare delivery [14]. In addition, prior studies showed that mobile assistive apps could improve the quality of life (QOL) for PLwD by assisting with tasks and encouraging independence, thus alleviating the caregiving burden [15,16,17]. 

Similarly, access to smartphones and other smart devices has grown exponentially across the globe. In 2018, it was estimated that 83% of adults in developing countries worldwide owned a smartphone [18]. In the same year, Malaysia had 17.2 million smartphone users, with a forecast of over 21 million by 2023 [19,20]. Given that the ownership of a smart device is not restricted to the upper-income groups [20], mobile apps that are used for health-related purposes have the potential to reach caregivers from underserved and marginalized populations [21]. Furthermore, the recent COVID-19 pandemic has boosted the use of mobile health apps [22], and more people worldwide are increasingly using apps to improve their health either by accessing information or utilizing health-related services [23,24,25]. 

### 1.2. Study Rationale

Evidence shows that many mobile apps developed for healthcare intervention and health improvements are highly effective [26,27,28,29]. Similarly, most dementia care mobile apps have been demonstrated to improve the QOL of PLwD and their caregivers and reduce caregiver burden [30,31,32]. Since the QOL of PLwD is intricately bounded to the QOL of their caregivers, apps that target caregivers as users are critical to strengthening dementia care in the community. Nevertheless, existing mobile apps for dementia are mostly developed in high-income regions [30,31,32,33] and, therefore, primarily and originally intended for their populations. In addition, more of these apps are designed for PLwD [34,35,36] instead of caregivers. As a result, while having excellent features, these apps are often not socio-culturally compatible with users or caregivers in other settings. For instance, existing apps tend to be in the English language [32,34,35,36,37], some incur high charges for services offered [21,32,38], and do not include aspects of caregiving that are unique to the local customs or social norms [38]. Thus, it is difficult to find a mobile app that meets the needs of PLwD caregivers in a specific context, in the low- and middle-income region [31,33]. Our in-depth literature review on dementia caregiving and existing mobile apps for PLwD caregivers identified a need for an app to support caregivers in the Malaysian context. 

### 1.3. Research Objectives

The general aim of this study is to develop and assess the usability of “Demensia KITA”, a mobile app for dementia caregivers in Malaysia. Specific objectives include:

- To develop suitable and relevant content for the mobile application.
- To develop and design a mobile app prototype that uses the national language (Bahasa Malaysia) as the interface.
- To determine the usability of the mobile app among PLwD caregivers.

## 2. Methodology

### Study Design

This research employs a combination of qualitative and quantitative methods and comprises three phases. In Phase I, Focus Group Discussion (FGD) and Nominal Group Technique (NGT) will be used to develop the app content. In Phase II, the mobile app is developed and designed. In Phase III, the usability of the app is tested using the Malay version of the mHealth App Usability Questionnaire (M-MAUQ). 

**Phase I: Establishment of an expert committee for developing the app content.**

Firstly, an expert committee consisting of geriatricians, geriatric psychiatrists, occupational therapists, dietitians, dentists, nurses, and caregivers will be established. These various types of expertise were determined based on discussions with two public health experts and a geriatric psychiatrist from Universiti Teknologi MARA (UiTM), who have previously worked on several dementia-related projects. This phase is expected to take approximately three months to complete. The main objective is to gather and discuss the relevant content that should be included in the app. This will be conducted using FGDs among the experts, and NGT among the PLwD caregivers. The NGT will be conducted first to gather information about the needs of PLwD caregivers and later communicate that knowledge to the group of experts.

**1.**  **Nominal Group Technique (NGT)**

In this study, NGT is aimed at PLwD caregivers. NGT refers to a structured variation of a small-group discussion to achieve consensus from the target groups most closely related to the area of concern. It involves four steps: generating, recording, discussing, and voting on ideas [39]. Depending on the participant’s availability, NGT will be conducted face-to-face or online using video conference platforms, such as Google Meet [40].

**NGT sampling and selection criteria:**

While NGT has been conducted with groups of between 2 and 14 people, a limit of seven has been recommended [41,42]. Seven caregivers of PLwD will be purposively selected from two tertiary healthcare institutions in Malaysia: University Malaya Medical Centre (UMMC) and General Hospital Kuala Lumpur (GHKL), as they serve a greater number of PLwD than other institutions in Malaysia and have well-established multidisciplinary teams for the management of PLwD. To ensure the groups’ generalizability, different caregivers from diverse backgrounds will be selected (i.e., different social statuses, different age groups, different education levels, and different ethnicity). Individuals who have not consented and are not able to communicate independently in English or Malay language will be excluded from the study. The following criteria apply to NGT participants:

Inclusion criteria for caregivers:

- Primary caregiver of a person living with mild dementia. A primary caregiver is defined as a person who helps in the personal care of an older adult with dementia, regardless of their education or experience, for one month or more, in the last 12 months [43,44].
- Aged ≥ 18 years old.
- Primary caregiver of a dependent person aged ≥ 60 years old diagnosed with any form of dementia.

All participants will be briefed about the NGT approach. First, the moderator will deliver the questions or problems to the group (in written form) and read them. She will then instruct everyone to write down their thoughts in short phrases or statements, discreetly and independently for 20 min [41]. Second, members of the group give each other comments in a round-robin approach without debate at this point. An idea from a group member is written on a whiteboard or chart visible to the whole group, and this process will continue until all points are recorded. Third, each idea is discussed for clarity and relevance. This stage allows group members to convey their feedback and opinion regarding the idea given in terms of rationale and value. Fourth, each person will rank the compiled ideas privately, then vote to determine which points should be prioritized. An example to illustrate this process is as follows: each group member selects five items from the group list and writes one on a card or paper. Next, each member ranks the five ideas, with the most important receiving a score of 5 and the least important receiving a score of 1 [45]. Examples are shown in Table 1:

Next is the determination of the NGT threshold, which refers to the value of the NGT score above which solution is considered relevant and should be prioritized for execution [45]. Generally, the NGT threshold should be half the maximum of the NGT score [45]. In the preceding example, the maximum possible NGT score of 5 × 5 is 25. The NGT threshold is half the maximum attainable NGT score (12.5) or scores equal to or above 13. Therefore, solutions or ideas 1, 3, 4, and 5 should be prioritized for implementation. 

For an online NGT, participants will be informed beforehand of their roles, as traditional procedural parts of meetings have been limited by screen-based engagement [40]. The group will be given approximately 30 min to ensure that everyone has an opportunity to practice utilizing the virtual platform. A similar method will then be used, except that when group members generate ideas, they will write them down on a piece of paper and show them on screen or read them out loud, while the moderator records their ideas on a PowerPoint presentation shared with everyone in real time.

**2.**  **Focus Group Discussion (FGD)**

FGD is a group interview with approximately 6 to 12 individuals who share similar qualities or interests. This technique is beneficial for acquiring quicker, in-depth insight into a subject that is more difficult to understand (or acquire data) using other methods [46]. The FGD involves field experts going through a series of topics to exchange ideas and points of view, aiming to reach a consensus on app themes and appropriate content [46]. Findings from the NGT sessions will be discussed with FGD participants. FGD participants will consist of geriatricians, geriatric psychiatrists, occupational therapists, nurses, dietitians, and dentists. At least one member from each specialty will be identified and recruited to ensure diverse experiences and perspectives.

The FGD will be conducted face-to-face or via an online platform, depending on the circumstances and participants’ preferences [46]. If it is conducted online, all participants will first be briefed about the FGD approach, the session objectives, and the anticipated outcome. This step will take approximately 90 min [46]. Next, the facilitator will pose questions to the group and allow members to respond to each other’s views. Once consensus is reached, the moderator will compile all relevant information, summarize the findings, and communicate them to the expert committee for final approval. The content will then be converted into a format that suits a mobile app. 

**Phase II: Designing of “Demensia KITA”**

Phase II will begin upon completion of phase I. It will entail designing and developing the mobile app, named “Demensia KITA”, with a software developer from within academia specializing in health-related mobile apps.

The preliminary prototype in this phase will be a native mobile application [47] optimized for a specific device and operating system and can be downloaded and installed from a web store (Play Store). It will be free of charge at this stage. Android will be selected as the mobile operating system due to its global dominance [48]. Apart from appropriate typography [49], the prototype aims to incorporate four primary features: rich experience; intuitive; personalized experience; and simplicity [50]. According to Forbes, 14% of smartphone users delete an app that is difficult to use [50]. As a result, easy navigation and overall usability, such as fewer clicks and actions, will be considered to ensure a positive mobile app user experience. The user interface will be as straightforward and intuitive as possible. The prototype version of the mobile app will also function offline, potentially extending reach to underserved populations as internet access is only required for app downloading and software updating. 

Demensia KITA will be fully operated in *Bahasa Malaysia*. The registration process will be personalized and simplified, and users will be able to log in using their personal external accounts such as Google, Facebook, and Twitter. Apart from credible information regarding dementia care, there will be features such as relevant spiritual and cultural practices to keep caregivers motivated and engaged.

Phase II is expected to take approximately one year. The early prototype version will be registered for a one-time fee on Google’s Android (Google Play Store) operating system platform to evaluate its usability. The Alpha version (an early pre-release version that is part of a dedicated testing process) [51] will be tested among 30 dementia caregivers conveniently selected from GHKL and UMMC beforehand to ensure the functionality of the app.

**Phase III: Assessment of mobile app usability**

Phase III will involve the release of a Beta version (completion of the app’s development) [51] once corrections of the Alpha version are completed. The Beta version testing will be conducted on eligible participants to inform the app’s usability [52]. This phase will take approximately three months. The researcher will provide continuous feedback to the mobile app developer, and regular meetings will be held until a consensus is reached. Findings from Phase III will be used to provide input to the mobile app developer for modifications before the final prototype is released. 

**Study setting**

Phase III will be conducted at the Geriatric Department of UMMC and the Memory Clinic of GHKL. The estimated time frame is approximately three months.

**Study population and sampling procedure**

The target population in this phase is PLwD caregivers. We will recruit caregivers of PLwD who attend regular follow-ups at UMMC and GHKL. Prior to recruitment, the researcher will contact the healthcare staff at the selected institutions to estimate the total number of patients and their caregivers. If the amount is large enough to allow random sampling, this method will be done to select respondents. However, convenient sampling may be employed if the number is too small to allow for adequate probability sampling. The inclusion and exclusion criteria for caregivers are presented in Table 2.

**Sample size calculation**

The sample-to-item ratio of 5:1 will be used [53]. Since the M-MAUQ scale consists of 18 items, a total of 90 participants will be needed. Previous similar studies have also shown a comparable sample size [32,54,55]. With a potential non-response rate of 20%, approximately 108 individuals will be invited to join the study.

**Data collection**

After obtaining verbal and written consent from each respondent, the researcher will instruct participants to download the mobile app into their smartphones. Respondents are given four to six weeks to test and use the mobile app. Subsequently, the researcher will administer an online questionnaire via e-mail or WhatsApp containing the usability scale for assessment. 

**Variables and tools**

The usability of a mobile app is defined as the quality attribute that assesses how easy a system interface is for users [56]. Usability generally includes three domains: ease of use, interface and satisfaction, and usefulness. The *ease of use* domain in this study is defined as the extent to which understanding, learning, and operating the mobile app’s specific system or technology is free of physical and mental effort [57,58]. The *interface and user satisfaction* are linked to the convenience of obtaining quality information through the mobile app [59]. Lastly, *usefulness* is defined as the quality of having utility (helpfulness of the mobile app) in serving the intended purpose [60]. 

Usability is measured using the M-MAUQ scale [61]. The mHealth App Usability Questionnaire (MAUQ) was originally developed by Zhou and colleagues to evaluate the usability of mobile health applications among patients and healthcare providers [62]. It came in four versions that assess the type of app (interactive or standalone) and intended audience (patient or health care provider). For this study, the phrase “MAUQ standalone used by patient” is used. The term “patient” here refers to an individual who utilizes a mHealth application to monitor, improve, or manage their health [61,62]. According to Zhou, the questionnaire comprises 18 items and is divided into three subscales: ease of use (five items), interface and satisfaction (seven items), and usefulness (six items). Participants rate each item on a seven-point Likert scale ranging from 1 (strongly disagree) to 7 (strongly agree). App usability is determined by the sum and average of all statements—the higher the average, the better. However, if the average score is less than 4, the app’s usability is poor [61]. The highest possible score is 126, and the lowest is 18. An average score equal to, or greater than 72 indicates that the app is usable. The M-MAUQ questionnaire has been translated into the Malay language and validated among the Malaysian population with a Cronbach alpha of 0.94 [61]. Its content validity indexes (CVI) for relevance and clarity were 0.98 and 0.94, respectively, and the face validity index for understandability was 0.96. For each item in M-MAUQ, the kappa statistic indicated high agreement between raters, ranging from 0.76 to 1.09 [61]. 

For further clarification, we provide the operational definition and scoring system of the variables of interest in Phase III (usability) and its domains. Table 3 (below) shows the operational definition of usability and its domains.

**Methods of data management**

Phase I data management will follow the guidelines recommended by Knight (2018) for interviews and FGDs [63]. First, a digital voice recorder will be used to record the entire session if the session is conducted on-site. Meanwhile, if the session is virtual, a screen or meeting recording with encryption in the platform will be used. Next, the audio recordings and other data collected will be labeled with the date, location, and name of the participant and kept by the researcher for analysis. Finally, all participants will be informed about how the data are collected, managed, and used.

In Phase III, data from the Google form will be transferred into an Excel file (Version 2202), which is used for data cleaning and coding. We expect and aim for no missing data, and this is ensured by configuring the Google form’s settings to mandate participants to answer all questions. Data will be verified before importing into the IBM Statistical Package for Social Sciences (SPSS) software version 26.0 for analyses. 

All data will be password protected and accessible only to the main researcher(s). They will also remain anonymous, and confidentiality will be ensured. Upon completion of the study, the Google form link will be disabled, and no other person except for the researchers will be able to access the information. All data in the Google cloud storage will be deleted or inactivated five years from the final publication date [64].

**Ethics Approval**

This study has been approved by the Malaysian Research and Ethics Committee (MREC), which serves as the principal National Medical Research Registry (NMRR) in Malaysia, Registration ID-22-00546-EBB (IIR) and the institute; University Teknologi MARA (UiTM) (Reference number: REC/07/2022 PG/MR/153). The study will be conducted in accordance with National Institutes of Health (NIH) standards [65]. Any modifications to the procedures stated in this protocol will be forwarded to the Malaysian Research and Ethics Committee (MREC), which serves as the principal National Medical Research Registry (NMRR) in Malaysia and explained in subsequent publications. 

**Figure 1:**
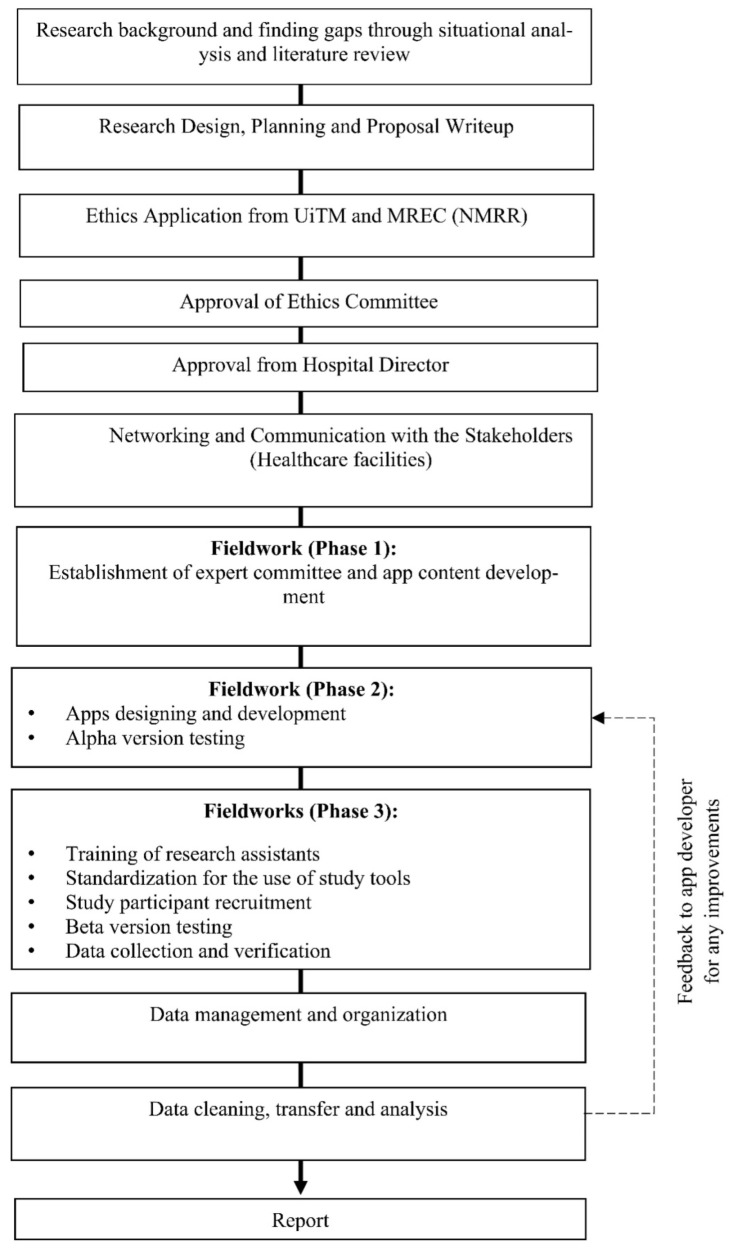
Overall study flowchart.

The overall flow of the study is demonstrated in Figure 1 below. A continuous literature review will be conducted to stay on track and be informed about the most recent information available.

## 3. Expected Results

**i.**  **Phase I:**

Even though the final content will be determined based on the FGD with the experts and NGT with dementia caregivers, we expect several topics or themes to emerge from the discussions [38]. Examples are not restricted to but may include:

(a) Educational materials in a written form (i.e., behavioral, and psychological symptoms, pharmaceutical side effects, nutritional and oral health, and disease progression or complications).
(b) Effective communication abilities (communication strategies or sharing experiences with other caregivers)
(c) Reminders (e.g., medication diary and appointment with a physician for PLwD)
(d) Psycho-spiritual assistance (e.g., a list of prayers or motivational quotes that are suited to the Malaysian cultural and religious ethos.)
(e) Access to nearby healthcare facilities or local community resources (a directory of hospitals, geriatric units, dementia centers, non-governmental organizations, police, and fire stations)

The scores from FGD will be presented in mean and standard deviation, and results will be determined according to the NGT threshold [45]. FGD results will be guided by thematic analyses [66].

**ii.**  **Phase II:**

An offline mobile app prototype called “Demensia KITA” in *Bahasa Malaysia* is user-friendly, culturally appropriate for local PLwD caregivers, and available in the Android Google Play Store, free of charge.

**iii.**  **Phase III:**

**Mobile app usability**

The total mean scores for usability from the M-MAUQ will be calculated and expressed as mean and standard deviation (SD) and tabulated. A total mean score equal to, or more than, 72 indicates that the app has good usability and vice versa. The mean score of each domain in the M-MAUQ (i.e., ease of use, usefulness, interface, and satisfaction) will be calculated. A cut-off score of ≥4 from each item in the domain indicates good usability. 

## 4. Discussion

Dementia has a complex and multifaceted impact on PLwDs and their caregivers. Unfortunately, in Malaysia, there is a relative lack of awareness and understanding about dementia, which has resulted in stigma and thus created barriers to diagnosis and treatment [2]. This study aims to develop a mobile app to support PLwD caregivers by providing information, facilitating access to nearby health services, maximizing the use of available local resources, and improving caregiving skills. To the best of our knowledge, this is the first mobile app in *Bahasa Malaysia* specifically designed for PLwD caregivers in the local context. 

The recent COVID-19 pandemic fosters digital transformation and adoption across multiple industries and sectors [67]. Evidence shows that the pandemic has increased the global uptake of mobile health apps [22], with more individuals, including older adults, encouraged to improve their health [23]. In the case of dementia, numerous memory clinics have been forced to cease operations due to the COVID-19 pandemic, prompting experts to call for global support for PLwD care [68]. 

We anticipate several challenges while implementing this study. In Phase I, careful planning is crucial for us to complete it within a specific time frame. Scheduling FGDs with clinicians and other field experts can be daunting due to their busy schedules, especially if we plan to conduct a physical session. We foresee the possibility of having to send repeated reminders or conducting one-on-one sessions for those unable to attend the group sessions. Similarly, we need to strike a balance when opting for online or on-site sessions; each has its advantages and disadvantages. NGTs and FGDs also require good communication skills on the moderator’s part to maximize the benefit of the sessions. For instance, the moderator (researcher) needs to know how to handle different group dynamics, prompt participants (to give more feedback), ensure that everybody is heard, and manage disputes or arguments when they occur [39,46,69]. Understandably, these are not easy tasks, if not handled well, they can hamper the data collection process. Likewise, the moderator must exercise caution while asking for feedback and ideas; if there are too many, it may be difficult to screen and narrow down the important and relevant ones. 

The phase of designing and developing the mobile app entails a set of challenges in the intersection between health and digital technology (and its implications)—some of which may not be familiar to healthcare providers and health researchers. For instance, when dealing with any software developer, it is critical to ensure that they are credible, as evidenced by previous successful projects, and capable of meeting the researcher’s requirements for the intended outcome to be achieved [70]. Additionally, it is important not to overlook legislative involvements to safeguard the interests, terms, conditions, and rights of all parties involved, including the researcher, the software developer, and the users [71,72]. This entails a fair agreement on copyrights and intellectual property ownership, data privacy, and how the app should be maintained and used after the completion of the study. Another issue that may arise is the commercialization of the mobile app once it is launched or after the study period. It is incumbent upon the researchers to take this into account in the planning stage and have a clear stand when discussing and negotiating with the software developer. Under normal circumstances, software developer companies are profit-driven business entities; thus, they are likely to have an interest in commercializing the mobile app. 

Researchers, on the other hand, may have different views and values, often with a strong desire to ensure equity and affordability while providing services. Such a discrepancy can sometimes result in tension or disputes. Nevertheless, these “conflicts” are manageable if both parties have a realistic expectation and understanding of each other’s role, values, and interests from the beginning and remain in constant effective communication that ensures a win-win situation. 

Of equal importance is mobile app sustainability in the long run, which again, needs to be part of the study plan. Engaging with a credible mobile app developer is one way of ensuring the utility and longevity of the app, but this requires balancing with other issues like ensuring affordable access and data privacy protection. Collaborations with private or business entities have their own merit but at times can get rather complicated for researchers to handle. In such cases, it is wise for the researcher to obtain legal advice from the legal unit of the academic institution or find alternatives, such as partnering with non-profit entities or software developers from within the academia. 

Another challenge is improving the generalizability of Phase III findings. If the study population is small—meaning the number of PLwD and their caregivers is not practically adequate to allow for a random selection—we may have to employ convenient sampling. However, this method is susceptible to selection bias, as the caregivers selected may not represent the whole caregiver population. To address this issue, universal sampling can be done. Alternatively, we can take additional steps to ensure a diverse sample regarding sociodemographic characteristics (e.g., age, race, education level, and location (urban or rural). 

## 5. Limitations and Strengths

This study has several limitations. First, convenient sampling may render selection bias as discussed earlier, given that our sample is derived from health centers in highly urbanized areas. Second, it is difficult to ensure that caregivers utilize the app frequently (during the trial period) before we assess its usability. However, this can be compensated by regular communication and encouragement. 

On the other hand, the study’s strengths include combining qualitative and quantitative methods with a relatively rigorous process of content development in Phase I. Similarly, this study can be considered unique as it brings together health, caregiving, and digital technology into one sphere—a complex combination that provides rich insight into the future of health research and pushes the boundaries of the roles of a typical health researcher. 

## 6. Conclusions

This protocol describes and elaborates on the steps and processes of developing a mobile app for PLwD caregivers in the Malaysian context. Our findings will contribute to the existing body of knowledge on the interaction between digital health modalities—specifically mobile apps—with caregiving, aging, health, and well-being. Other than that, the study findings will highlight the needs of PLwD caregivers in the local context. The mobile application is expected to be launched and made available to the public through collaborations with other stakeholders, such as senior citizen advocacy groups and the Malaysia Ministry of Health. These partnerships can accelerate the adoption of digital health and technologies in caregiving at the policy level. In addition, the availability of a caregiver mobile app can aid clinicians in managing PLwD by providing caregiver support. 

For future research, we recommend a study to test the effectiveness of this mobile app in reducing caregiver burden and mental stress and improving the QOL of PLwD caregivers. This can be conducted using intervention/experimental designs such as randomized controlled trials or quasi-experimental studies. Another potential research area is the replication of this mobile app in another setting, with some adjustments and modifications to the app. More importantly, we hope to holistically contribute to the betterment of PLwD caregivers by making this tool (app) available and prompting more uptake of digital technology in the field of caregiving while ensuring equity and sustainability.

## Figures and Tables

**Table 1:** Example of Nominal Group Technique Voting Scores Template.

Solution/Ideas	Participant 1	Participant 2	Participant 3	Participant 4	Participant 5	NGT Scores
Solution/Idea 1	4	4	5	4	5	22
Solution/Idea 2	2	1	3	2	2	10
Solution/Idea 3	5	5	5	4	4	23
Solution/Idea 4	3	1	5	2	4	15
Solution/Idea 5	4	4	5	4	3	20

**Table 2:** Inclusion and exclusion criteria in Phase III.

Criteria	Eligibility
**Inclusion**	Primary caregiver of a person living with dementia. A primary caregiver in this study is defined as a person who helps in the personal care of an older adult with dementia, regardless of their education or experience for one month or more during the last 12 months [43,44]. Aged ≥ 18 years old. Caring for an older relative aged ≥ 60 years old diagnosed with any form of dementia. Owns a working and compatible smartphone (Android with mobile data). Has basic knowledge of using smartphones and apps.
**Exclusion**	Not able to communicate independently in English or Bahasa Malaysia. Does not use Android as their mobile operating system.

**Table 3:** Operational definition of usability and its domains.

Variable/Domain	Operational Definition
**Usability of mobile app**	Usability is determined by the total and mean scores of all domains (ease of use, interface, and usefulness) derived from the M-MAUQ. Higher scores indicate greater usability. A total mean score equal to, or more than, 72 denotes that the app has good usability and vice versa.
**(a) Ease of use**	There are 5 items under this domain. A total mean score equal to, or more than, 20 under indicates that the app is easy to use, and vice versa.
**(b) Interface and satisfaction**	There are 7 items under this domain. A total mean score equal to, or more than, 28 indicates that the app has a good interface and is satisfactory, and vice versa.
**(c) Usefulness**	There are 6 items under this domain. A total mean score equal to, or more than, 24 indicates that the app is useful and serves its purpose and vice versa.

## Data Availability

The data presented in this study will be available on request from the corresponding author.
